# Prospective multicenter study on the incidence of surgical site infection after emergency abdominal surgery in China

**DOI:** 10.1038/s41598-021-87392-8

**Published:** 2021-04-08

**Authors:** Ze Li, Hui Li, Pin Lv, Xingang Peng, Changliang Wu, Jianan Ren, Peige Wang

**Affiliations:** 1 Department of Emergency Medicine, The Affiliated Hospital of Qingdao University, 16 Jiangsu Road, Qingdao, 266000 People’s Republic of China; 2 Department of Pharmacy, Qingdao Mental Health Center, Qingdao University, 299 Nanjing Road, Qingdao, 266000 People’s Republic of China; 3 Lab for Trauma and Surgical Infections, Department of Surgery, Jinling Hospital, Affiliated To Southeast University, 305 East Zhongshan Road, Nanjing, 210002 People’s Republic of China

**Keywords:** Diseases, Health care, Risk factors

## Abstract

There is still a lack of relevant studies on surgical site infection (SSI) after emergency abdominal surgery (EAS) in China. This study aims to understand the incidence of SSI after EAS in China and discuss its risk factors. All adult patients who underwent EAS in 47 hospitals in China from May 1 to 31, 2018, and from May 1 to June 7, 2019, were enrolled in this study. The basic information, perioperative data, and microbial culture results of infected incision were prospectively collected. The primary outcome measure was the incidence of SSI after EAS, and the secondary outcome variables were postoperative length of stay, ICU admission rate, ICU length of stay, 30-day postoperative mortality, and hospitalization cost. Univariate and multivariate logistic regression were used to analyze the risk factors. The results were expressed as the odds ratio and 95% confidence interval. A total of 953 patients [age 48.8 (SD: 17.9), male 51.9%] with EAS were included in this study: 71 patients (7.5%) developed SSI after surgery. The main pathogen of SSI was *Escherichia coli* (culture positive rate 29.6%). Patients with SSI had significantly longer overall hospital (*p* < 0.001) and ICU stays (*p* < 0.001), significantly higher ICU admissions (*p* < 0.001), and medical costs (*p* < 0.001) than patients without SSI. Multivariate logistic regression analysis showed that male (*P* = 0.010), high blood glucose level (*P* < 0.001), colorectal surgery (*P* < 0.001), intestinal obstruction (*P* = 0.045) and surgical duration (*P* = 0.007) were risk factors for SSI, whereas laparoscopic surgery (*P* < 0.001) was a protective factor. This study found a high incidence of SSI after EAS in China. The occurrence of SSI prolongs the patient's hospital stay and increases the medical burden. The study also revealed predictors of SSI after EAS and provides a basis for the development of norms for the prevention of surgical site infection after emergency abdominal surgery.

## Introduction

The WHO states in the surgical site infection prevention guideline^1^: Surgical site infection (SSI) is among the most common health-care-associated infections in developing countries. The occurrence of SSI causes great pain to patients, prolongs the length of hospital stay, and causes expensive hospitalization cost^2,3^.

Current studies have found that the incidence of SSI after abdominal surgery ranges from 1.2 to 5.2%^4–6^. The incidence of SSI is much higher in patients undergoing emergency abdominal surgery (EAS) than in elective surgery^7,8^. A cross-sectional study in the United States has shown that the incidence of incisional SSI in EAS patients is 6.7%, however, the respective incidence and proportion of drug-resistant bacteria in China are higher^9,10^. In recent years, more patients undergo EAS with the rising number of emergency cases, patient management is more centralized, and the prevention of SSI has become more important^11^. However, there are limited studies on SSI after EAS in China. Therefore, it is important to obtain relevant data about SSI after EAS in China and provide a basis for its prevention.

In this study, prospective clinical data was collected from EAS patients in 47 hospitals in China, and the associated risk factors were analyzed. This study aims to describe the incidence of SSI in EAS and the related risk factors. Further, it provide the necessary evidence for the prevention of SSI after EAS.

## Methods

We conducted a multicenter, prospective, cross sectional study. The demographic and perioperative characteristics were collected to further evaluate the rate, risk factors and microbiological profile of SSI after EAS in China. Sample size estimation was 826 or larger, which is the sample size required to estimate the incidence, given α = 0.05, δ = 0.02, expected incidence = 7.0%, and lost to follow-up rate = 20%.

All adult patients (over 18 years) who underwent EAS in 47 tertiary hospitals in China from May 1, 2018, to May 31, 2018, and from May 1, 2019, to June 7, 2019, were included (surgical indications are shown in Table S1). The follow-up period was 30 days postoperatively. Patients who were participating in other clinical trials, pregnant, undergoing urologic, gynecologic, or transplant surgery were excluded. This study has been approved by the ethics committee of Jinling Hospital (Approval No.: 2018NZKY-002-01). Written informed consent was obtained from all study participants. All methods were carried out in accordance with relevant guidelines and regulations.

Data related to SSI was collected according to established study protocols. Patient baseline variables included: Age, sex, body mass index (BMI), admission diagnosis, American Society of Anesthesiologists (ASA) physical status score, hypertension, diabetes mellitus, chronic hepatic dysfunction (hepatitis, cirrhosis, and/or abnormal liver enzymes levels), chronic renal dysfunction (renal failure and/or dialysis), chronic cardiac dysfunction (heart failure, myocardial infarction, previous percutaneous coronary intervention, and/or previous cardiac surgery), immune suppression status, smoking status (current smoker, former smoker, or nonsmoker), and preoperative concentrations of hemoglobin, albumin, and blood glucose(6 h before starting the operation). Surgery-related variables included type of surgery based on surgical site (colorectal or non-colorectal), surgical wound class (clean-contaminated, contaminated, or dirty), methods of bowel preparation (mechanical bowel preparation [MBP] or oral antibiotic bowel preparation [OABP]), hand preparation (disinfectant or scrubbing), use of laparoscopy or robotic surgery, use of incisional protection devices (gauze, adhesive drapes, wound edge protector, or something else), grade of lead surgeon (based on their title), duration of surgery (from incision to suture), and the national nosocomial infections surveillance (NNIS) risk index. The NNIS risk index ranged from 0 to 3 per the assessment of three variables: ASA score, surgical wound class, and duration of surgery; the cutoff values for each variable were an ASA score of 3, a contaminated surgical incision, and an operative time of 180 min, with 1 point assigned when each variable exceeded its respective cutoff value^12^. Antibiotic-related variables included the use of preoperative and postoperative antibiotics.

The data collected consisted of three validation steps. First, each hospital identified patients according to the study protocol, collected basic data, and followed up the patients. Second, three independent investigators (ZL, HL, and PW) collated all data to assess its accuracy. Third, team members discussed the problem data and qualitatively assessed the collection process and data.

The primary outcome was the rate of SSI at 30 days; patients discharged from the hospital were followed up at the outpatient office or by telephone. The secondary outcome variables were postoperative hospital stay, ICU admission rate, ICU length of stay, hospitalization cost (includes cost of patient readmission for surgical site infection), and 30-day mortality. Surgical site infections were classified according to the Centers for Disease Control and Prevention (CDC) criteria; superficial incisional, deep incisional, and organ or space infections^13^. The diagnosed of SSI was by the presence of the following: (1) Local pain or tenderness, local swelling, redness, heat, or several of these symptoms, combined with deliberate or spontaneous incision dehiscence; (2) drainage at the incision or drainage tube; (3) imaging diagnosis of intra-incision abscess or abdominal and pelvic infections (including anastomotic leak). The fluid from the drain or puncture where SSI occurred were cultured according to the standards of each hospital.

### Informed consent

This study was done in accordance with the strengthening the reporting of observational studies in epidemiology (STROBE) statement. Informed consent was obtained from all subjects including ICU patient and for died patients informed consent from a parent and/or legal guardian.

## Statistical analysis

Categorical variables between the two groups were compared using the χ2 test, adjusted χ2 test, or Fisher's exact test. Continuous variables were not normally distributed, and they were expressed as median (interquartile range) and compared using the Mann–Whitney U test. The significance level was set at the conventional level of α = 0.05. *P* values were two-sided, and *P* < 0.05 was considered statistically significant.

Univariate and multivariate logistic regression analyses were used to identify predictors of SSI occurrence. Univariate regression analysis was performed on the data first, followed by multivariate regression analysis on the statistically significant data. A backward stepwise regression procedure was used. In which continuous variables were substituted into the model by obtaining cut-off values through the maximum of the area under the ROC curve (i.e., the higher sensitivity and specificity). The variables related to the incidence of SSI were divided into categories: age (> 47.5 years or ≤ 47.5 years), preoperative hemoglobin concentration (< 11 g/dL or ≥ 11 g/dL), preoperative albumin concentration (< 4.1 g/dL or ≥ 4.1 g/dL), and preoperative blood glucose concentration (> 124 mg/dL or ≤ 124 mg/dL). The operation time (> 120 min) was taken as cut-off value according to the fixed percentile of 75. The results were expressed as the odds ratio and 95% confidence interval. Data analysis was performed using SPSS 22.0 software.

## Result

**Figure 1:**
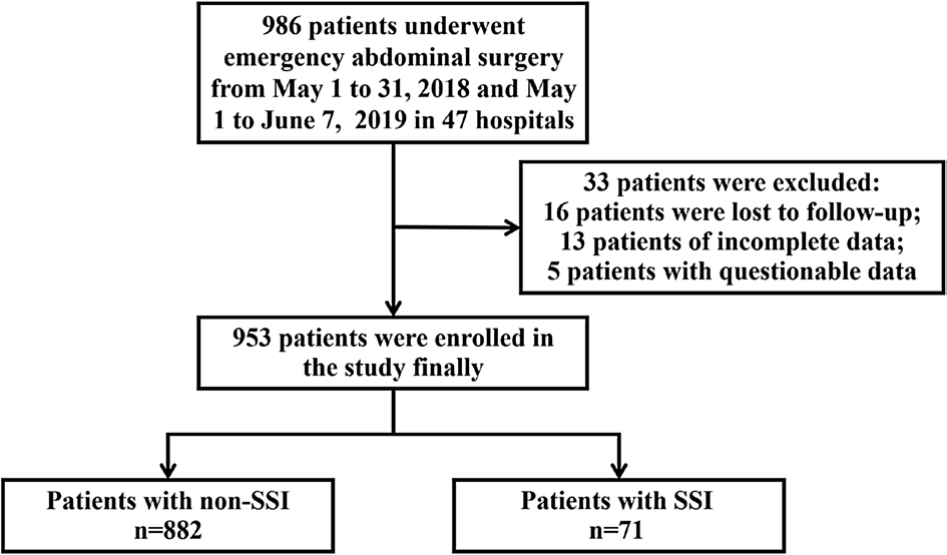
Patient flow chart.

The patient flow chart is provided in Fig. 1. The 953 cases (534 males and 419 females) that met the inclusion criteria were followed up and analyzed. The mean age of the patients was 48.8 (SD: 17.9), ranging from 18 to 88 years. Of the total, 216 patients had one or more co-morbidities, with hypertension and diabetes mellitus being the most common comorbidities (137, 57) (Table 1).

**Table 1:** Demographics of included patients.

Variables	SSI group (n = 71)	Non-SSI group (n = 882)	Statistic	*P* value
*Age, years, median (IQR)*	56 (48–70)	47 (33–63)	U = 39,918	< 0.001
*Gender (%)*			χ^2^ = 8.173	0.004
Male	51 (9.6)	483 (90.4)		
Female	20 (4.8)	399 (95.2)		
*BMI, Kg/m*^*2*^*, median (IQR)*	23.0 (22.0–26.0)	23.0 (22.0–24.4)	U = 20,738.5	0.695
*Comorbidity (%)*				
Diabetes mellitus	10 (17.5)	47 (82.5)	χ^2^ = 8.958	0.003
Hypertension	14 (10.2)	123 (89.8)	χ^2^ = 1.779	0.182
Chronic liver disease	1 (5.9)	16 (94.1)	χ^2^ = 0.021	0.885
Chronic kidney disease	3 (13.6)	19 (86.4)	χ^2^ = 0.500	0.479
Chronic heart disease	3 (9.4)	29 (90.6)	χ^2^ = 0.006	0.937
Tuberculosis	1 (12.5)	7 (87.5)		0.463
Steroid use	0	6 (100)		1.000
Immunosuppressive medication	0	2 (100)		1.000
*Smoking history (%)*			χ^2^ = 3.036	0.219
No	58 (7.2)	747 (92.8)		
Former	3 (18.8)	13 (81.2)		
Current	10 (7.6)	122 (92.4)		
*Hemoglobin, g/dL, median (IQR)*	12.5 (9.8–14.5)	13.1 (12.0–14.4)	U = 25,430.5	0.012
*Albumin, g/dL, median (IQR)*	3.6 (3.0–4.3)	4.0 (3.6–4.5)	U = 22,932.5	0.001
*Blood glucose, mg/dL, median (IQR)*	144 (106–199)	108 (93–128)	U = 43,743	< 0.001

Postoperative SSI was identified in 71 (7.5%) patients. The volume of patients with SSI, out of the total, in each participant hospital, is listed in Table S2. Among them, 23 patients (32.4%) had superficial incision infection, 27 (38.0%) had deep incision infection, and 21 (29.6%) had organ-space infection. Secretion and pus cultures were positive in 42 patients (59.2%); the pathogens were *Escherichia coli* in 21 patients (50.0%), *Klebsiella pneumoniae* in 7 patients (16.7%), *Staphylococcus aureus* in 6 patients (14.3%), *Enterococcus faecium* in 4 patients (9.5%), surface *staphylococci* in 2 patients (4.8%), and *Acinetobacter baumannii* and *Proteus mirabilis* in 1 patient each (2.4%). ICU admission and medical costs were significantly increased in the SSI group compared with the non-SSI group, and also, postoperative and ICU length of stay were significantly prolonged (Table 2). Four patients died within 30 days of surgery, but none was directly caused by SSI. The median duration from surgery to SSI was 5.2 d.

**Table 2:** Outcomes of included patients.

Variables	SSI group (n = 71)	Non-SSI group (n = 882)	Statistic	*P* value
Postoperative delivery of icu, n (%)	36 (50.7)	195 (22.1)	χ^2^ = 29.259	< 0.001
Length of ICU stay, day, median (IQR)	0 (0–2)	0 (0–0)	U = 41,004	< 0.001
Length of postoperative stay, day, median (IQR)	13 (8–19)	6 (4–9)	U = 49,486	< 0.001
Medical cost, thousand dollar, median (IQR)	6.9 (3.7–9.7)	2.3 (1.7–3.8)	U = 51,318	< 0.001
30-d mortality, n (%)	2 (3.0%)	2 (0.2%)		0.030

Demographic characteristics: There was a higher incidence of SSI in male than in female patients (*p* = 0.004), and in patients with diabetes mellitus than in those without it (*p* = 0.003). There was a statistically significant difference between the two groups in preoperative hemoglobin (*p* = 0.012), albumin (*p* = 0.001), and blood glucose concentrations (*p* = 0.000) (Table 1).

Perioperative characteristics: Colorectal surgery patients had a higher rate of SSI than non-colorectal patients (*p* < 0.001); those with additional intestinal obstruction, had a higher rate of SSI (*p* < 0.001). The ASA score (*p* < 0.001) and NNIS risk index (*p* < 0.001) were significantly different between the two groups. The incidence of SSI was significantly lower in patients who underwent laparoscopic surgery than for those who underwent laparotomy. Wound irrigation was significantly different between the groups (*p* < 0.001). The duration of EAS was significantly longer in the SSI group than in the non-SSI group (*p* < 0.001). Adhesive drapes were the most commonly used wound protectors in EAS. The lead EAS surgeon was usually a senior surgeon (Table 3).

**Table 3:** Perioperative characteristics of included patients.

Variables	SSI group (n = 71)	Non-SSI group (n = 882)	Statistic	P value
*Type of surgery (%)*			χ^2^ = 61.515	< 0.001
Gastric surgery	2 (2.8)	53 (6.0)		
Hepatobiliary surgery	4 (5.6)	56 (6.4)		
Small bowel surgery	17 (23.9)	100 (11.4)		
Appendix surgery	22 (31.0)	573 (65.1)		
Colon surgery	20 (28.2)	39 (4.4)		
Rectal surgery	2 (2.8)	3 (0.3)		
Others	5 (7.0)	69 (7.8)		
*Colorectal surgery (%)*			χ^2^ = 72.133	< 0.001
Yes	22 (31.0)	42 (4.8)		
No	49 (69.0)	840 (95.5)		
*Bowel preparation (%)*				0.528
None	62 (87.3)	778 (88.4)		
MBP only	4 (5.6)	27 (3.1)		
OABP only	5 (7.0)	75 (8.5)		
MBP + OABP	0	2 (0.2)		
*Intestinal obstruction (%)*			χ^2^ = 29.526	< 0.001
Yes	20 (28.2)	73 (8.3)		
No	51 (71.8)	809 (91.9)		
*Gastrointestinal perforation (%)*			χ^2^ = 0.031	0.861
Yes	6 (8.5)	80 (9.1)		
No	65 (91.5)	802 (91.1)		
*ASA score (%)*			χ^2^ = 31.630	< 0.001
1	15 (21.1)	340 (38.6)		
2	28 (39.4)	406 (46.1)		
3	20 (28.2)	112 (12.7)		
4	8 (11.3)	24 (2.7)		
*NNIS risk index*			χ^2^ = 91.767	< 0.001
0	17 (23.9)	551 (62.6)		
1	27 (38.0)	252 (28.6)		
2	19 (26.8)	73 (8.3)		
3	8 (11.3)	6 (0.7)		
*Approach*			χ^2^ = 53.600	< 0.001
Open	61 (85.9)	362 (41.1)		
Laparoscopic	10 (14.1)	520 (59.1)		
*Surgical wound class (%)*			χ^2^ = 0.766	0.381
Clean-contaminated	48 (67.6)	639 (72.6)		
Contaminated or dirty	23 (32.4)	243 (27.6)		
*Incisional protection (%)*			χ^2^ = 23.051	< 0.001
None	29 (40.8)	537 (61.0)		
Gauze	14 (19.7)	135 (15.3)		
Adhesive drapes	12 (16.9)	145 (16.5)		
Wound edge protector	16 (22.5)	65 (7.4)		
*Wound irrigation (%)*			χ^2^ = 23.442	< 0.001
No	12 (16.9)	406 (46.1)		
Saline	35 (49.3)	306 (34.8)		
Povidone iodine solution	24 (33.8)	170 (19.3)		
*Grade of lead surgeon (%)*			χ^2^ = 12.356	0.002
Junior	0	71 (8.1)		
Middle	22 (31.0)	371 (42.2)		
Senior	49 (69.0)	440 (50.0)		
*Surgical duration, min, median (IQR)*	130.0 (83.0–205.0)	75.0 (53.0–114.3)	U = 45,556.5	< 0.001

For antibiotic administration, a total of 707 patients received preoperative antibiotic. Third-generation cephalosporins were the most commonly used antibiotic type (32.8%), followed by cephamycins and combination antibiotic. Antibiotic were used postoperatively in 872 patients, with the most patients receiving combination antibiotic (31.8%), followed by the third-generation cephalosporins in 200 patients. Postoperative antibiotic use was significantly longer in the SSI group than in the non-SSI group (*p* < 0.001) (Table 4).

**Table 4:** Antibiotics use.

Variables	SSI group (n = 71)	Non-SSI group (n = 882)	Statistic	*P* value
*Perioperative prophylactic antibiotic (%)*				0.005
None	14 (19.7)	232 (26.4)		
2nd cephalosporin	14 (19.7)	70 (8.0)		
3rd cephalosporin	21 (29.6)	211 (24.0)		
Cephamycin	6 (8.5)	137 (15.6)		
Oxacephem	1 (1.4)	23 (2.6)		
Penicillin	2 (2.8)	70 (8.0)		
Aminoglycoside	0	3 (0.3)		
Quinolones	1 (1.4)	22 (2.5)		
Carbapenem	5 (7.0)	14 (1.6)		
Nitromidazole	2 (2.8)	13 (1.5)		
Other	0	4 (0.5)		
Combined antibiotic	5 (7.0)	83 (9.4)		
*Postoperative antibiotic (%)*				0.002
None	2 (2.8)	79 (9.0)		
2nd cephalosporin	6 (8.5)	81 (9.2)		
3rd cephalosporin	17 (23.9)	183 (20.8)		
Cephamycin	9 (12.7)	123 (14.0)		
Oxacephem	2 (2.8)	16 (1.8)		
Penicillin	5 (7.0)	68 (7.7)		
Aminoglycoside	1 (1.4)	2 (0.2)		
Quinolones	1 (1.4)	23 (2.6)		
Carbapenem	9 (12.7)	30 (3.4)		
Nitromidazole	1 (1.4)	0		
Other	3 (4.2)	15 (1.7)		
Combined antibiotic	15 (21.1)	262 (29.8)		
*Duration of postoperative antibiotic (%)*			χ^2^ = 19.359	< 0.001
1d	3 (4.2)	30 (3.4)		
2-4d	15 (21.1)	278 (31.6)		
5-7d	23 (32.4)	360 (40.9)		
> 7d	28 (39.4)	135 (15.3)		
Median (IQR)	7.0 (4.0–9.0)	5.0 (3.0–7.0)	U = 41,725	< 0.001

**Figure 2:**
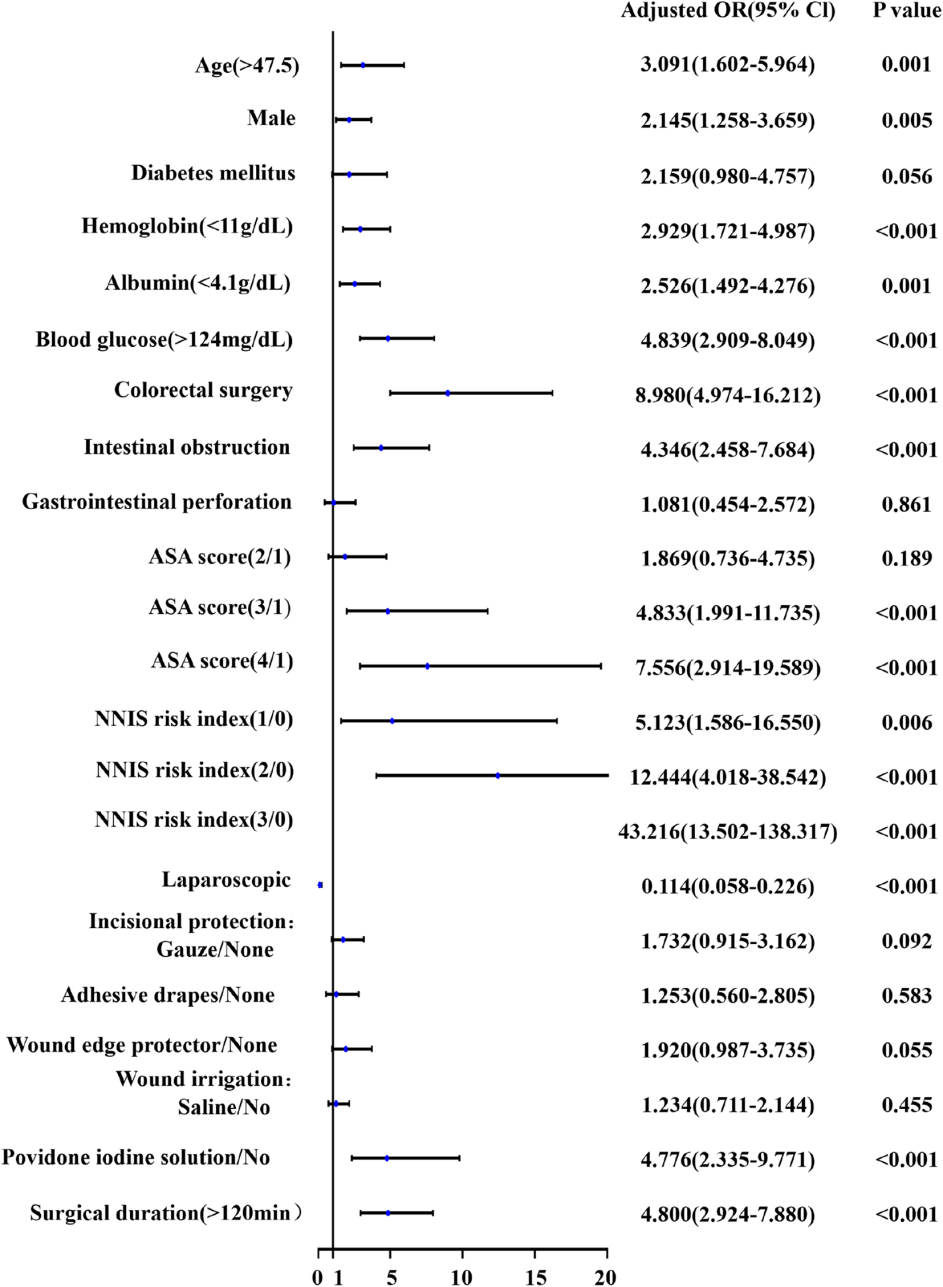
Results of univariate logistic regression analysis.

Univariate logistic regression analysis risk factors results for SSI are shown in Fig. 2. The factors significantly associated with the occurrence of SSI were age (> 47.5 years), male gender, preoperative hemoglobin concentration (< 11 g/dL), preoperative albumin concentration (< 4.1 g/dL), preoperative blood glucose concentration (> 124 mg/dL), colorectal surgery, intestinal obstruction, ASA score greater than 2, NNIS risk index greater than 0, laparoscopic surgery, wound irrigation (povidone iodine solution), and operation time (> 120 min). Multivariate logistic regression analysis confirmed that male were more likely to develop SSI compared with female (OR 2.203; 95% CI 1.204, 4.029; *p* = 0.010), patients with blood glucose concentrations > 124 mg/dL were more likely to develop SSI(OR 3.331; 95% CI 1.890, 5.872; *p* < 0.001), patients with colorectal surgery were more likely to develop SSI than patients with EAS at other sites (OR 7.031; 95% CI 3.579, 13.813; *p* < 0.001), patients with preoperative intestinal obstruction were more likely to develop SSI (OR 1.974; 95% CI 1.015, 3.839; *p* = 0.045), and patients with operation time > 120 min were more likely to develop SSI (OR 2.918; 95% CI 1.671, 5.096; *p* < 0.001). However, patients who underwent laparoscopic surgery had fewer SSI than those who underwent open surgery (OR 0.206; 95% CI 0.099, 0.430; *p* < 0.001) (Table 5).

**Table 5:** Results of multivariate logistic regression analysis.

Variables	B	OR	95%CI	Statistic	*P* value
Gender (male)	0.790	2.203	1.204–4.029	6.568	0.010
Blood glucose (> 124 mg/dL)	1.203	3.331	1.890–5.872	17.312	< 0.001
Colorectal surgery	1.950	7.031	3.579–13.813	32.048	< 0.001
Approach (Laparoscopic)	-1.580	0.206	0.099–0.430	17.740	< 0.001
Intestinal obstruction	0.680	1.974	1.015–3.839	4.011	0.045
Surgical duration (> 120 min)	1.071	2.918	1.671–5.096	14.164	< 0.001

## Discussion

This study found that the incidence of postoperative SSI in EAS patients was 7.5%, with *Escherichia coli* infection being the most common, which is comparable to that reported worldwide^7,9,14^. It has been shown that older people are more likely to develop SSI^15^; in this study, age was not a predictor as observed using multivariate logistic regression analysis. Male patients had a significantly higher risk of SSI after EAS than females; while this is partly debated, multiple studies have found significantly higher rates of SSI in male patients than in females^11,16^. This may be related to male bushy hair; shaving increases the risk of skin trauma. Guidelines for SSI prevention issued by the Surgical Infection Society strongly discourage the use of razors for hair removal and propose the use of clippers if necessary.

In our study, high blood glucose level (> 124 mg/dL) was found to be a risk factor for SSI after EAS. Previous studies have shown that diabetes mellitus and preoperative hypergycemia are SSI risk factors^17^. Hyperglycemia can affect the function of white blood cells, which in turn reduces the body's defense. The EAS patients tend to be in an acute preoperative stress state, and the associated hyperglycemic levels can better predict SSI than diabetes. Decreased serum albumin is usually an indicator of malnutrition or combined chronic wasting disease. In the present study, low serum albumin levels did not significantly affect the occurrence of SSI after adjustment for other variables, which requires further investigation.

The incidence of postoperative SSI is generally higher in patients undergoing colorectal than other gastrointestinal surgeries^18^. Our data shows that patients undergoing emergency colorectal surgery are at 7.017 times higher risk of SSI than patients undergoing other emergency gastrointestinal surgeries. There is a high colorectal bacterial load, including a variety of gram-negative and anaerobic bacteria. Necessary bowel preparation is difficult to achieve for emergency surgery and intestinal contents are easily spilled, contaminating the surgical area. This explains the high risk of SSI in emergency colorectal surgery patients.

Our study found that intestinal obstruction was a risk factor for SSI. The intestinal barrier function is impaired due to fasting and dilatation of the bowel lumen regardless of whether the bowel is removed, and bacteria are easily translocated outside the bowel lumen, increasing the risk of SSI. In our study, gastrointestinal perforation was not an independent risk factor for SSI. Gastrointestinal perforation usually occurs in the upper gastrointestinal tract, which has a relatively less bacterial load. At the same time, more attention is often paid to SSI prevention during the treatment of patients with gastrointestinal perforation, including adequate irrigation of the surgical site and post-surgical application of high-grade antibiotics. There is need to pay attention to the prevention of SSI in patients with intestinal obstruction regardless of whether bowel resection is performed or not.

Our study also evaluated the relationship of ASA score, NNIS risk index, and wound irrigation with SSI. None of these were independent risk factors for SSI in EAS patients after adjustment for logistic regression analysis. The NNIS risk index included ASA score, duration of surgery, and surgical wound grade. Since NNIS risk index includes ASA score, we performed multiple logistic regression analysis on the results that were statistically significant by univariate analysis after excluding NNIS risk index or ASA score, and the results were not significantly different. The majority of patients had an ASA score of 1 or 2, and surgical wounds were classified as clean-contaminated. The incidence of SSI was higher in patients whose incisions were irrigated with saline and povidone-iodine solution. This may be due to more contaminated incisions being irrigated than clean incisions during surgery, therefore spreading the infective microbes. The World Society for Emergency Surgery (WSES) states in the Intraoperative Surgical Site Infection Control and Prevention that there is insufficient data to support the role of irrigation of the incision with saline or polyvidone before closure in preventing SSI^19^.

Previous studies have shown that laparoscopic surgery can significantly reduce the incidence of SSI compared to open surgery^18,20^. This study supports this view. Laparoscopic surgery uses a small incision, greatly reducing the chances of exposure with little damage to the surrounding tissues, which reduces the risk of SSI. However, laparoscopic surgery has some limitations, especially for emergency cases. Laparoscopy requires certain equipment base, operating space, and experienced surgeons. In the 2016 WSES consensus on the management of intra-abdominal infections, laparoscopic surgery was determined to be safe and preferred for procedures such as appendectomy, repair of perforated peptic ulcer, and cholecystectomy when contraindications are excluded^21^. However, if peritonitis episodes were > 24 h, laparotomy was recommended.

The duration of surgery is a risk factor for SSI^22,23^. This study found that EAS patients who had longer surgery were more likely to have an SSI. Longer surgery not only aggravates the destruction of the microenvironment in the surgical area, but also greatly increases the chances of bacterial colonization due to the increased exposure time of the surgical incision to the air. Precautions against SSI can be appropriately instituted for operation time greater than 120 min.

We also analyzed patients' perioperative antibiotic use. The American College of Surgeons and Surgical Infection Society recommended administering prophylactic antibiotics only when indicated^24^. However, EAS patients are mostly treated for the primary disease in the emergency department before surgery; in our study 707 (74.2%) patients had received different classes of antibiotics, preoperatively. Meanwhile, the postoperative application time of antibiotics is 2–7 days. Although EAS patients are often complicated with intra-abdominal infection before operation, this also shows that there is lack of perioperative antibiotic standards for use in emergency surgery in China. Further randomized controlled trials are needed to determine the type and timing of antibiotic therapy. The WHO, American College of Surgeons and Surgical Infection Society recommend that the administration of antibiotics should not be prolonged after surgery^1,24^. Interestingly, we found that the high incidence of SSI was associated with prolonged antibiotic administration; EAS patients who were on prophylactic antibiotics, are usually continued on antibiotics post surgically. The study shows that prolonged prophylactic antibiotics use is not beneficial in reducing the incidence of SSI, but leads to intestinal flora disturbances, drug-resistant bacteria, and increased medical burden^25,26^.

## Limitations of the study

This study has several limitations. The study period is short and only includes patients who underwent EAS from May 1 to May 31, 2018, and May 1, June 7, 2019, which may have some bias. The study included different types of patients, which may be a confounding factor. Also, the results of bacterial culture of samples may be biased due to different standards and technical levels of culturing in each hospital. The multicenter study will be refined in the coming years.

## Conclusions

This study found a high incidence of SSI after EAS in China. The occurrence of SSI prolongs the patient’s hospital stay and increases the medical burden. We recommend that SSI should be monitored in real time nationwide, standards for the use of antibiotics after emergency abdominal surgery should be developed, and prevention and treatment strategies for surgical site infections should be continuously optimized. Minimize the occurrence of surgical site infections.

## Supplementary Information

Supplementary Legends.

Supplementary Table 1.

Supplementary Table 2.

## Data Availability

The datasets generated and analyzed during the current study are not publicly available. Because they are archived in the clinical databases of all participating hospital and the Affiliated Hospital of Qingdao University, they are only used for scientific purposes. Datasets are available from the corresponding author upon request.

## Abbreviations

ASA: American Society of Anesthesiologists
CDC: Center for Disease Control and Prevention
EAS: Center for Disease Control and Prevention
ICU: Intensive Care Unit
NNIS: National Healthcare Safety Network
PP mesh: Polypropyleneps mesh
SPSS: Statistical Package for Social Sciences
SSI: Surgical site infections
WHO: World Health Organization
WSES: World Society for Emergency Surgery
