# Supplementary material for: Prospective multicenter study on the incidence of surgical site infection after emergency abdominal surgery in China
Source: Sci Rep. 2021 Apr 8;11:7794. doi: 10.1038/s41598-021-87392-8 (PMC8032698; doi:10.1038/s41598-021-87392-8)
Supplement: Supplementary file 1 — Supplementary Legends. [file 41598_2021_87392_MOESM1_ESM.docx]

**Prospective** **Multicenter Study of Surgical Site Infection After Emergency Abdominal Surgery in China**

Ze Li, Hui Li, Pin Lv, Xingang Peng, Changliang Wu, Jianan Ren, Peige Wang

**Table S1 The indications for surgery**

**Table S2 Number of patients and number of patients with SSI per participating hospital**
