# Supplementary material for: Prospective multicenter study on the incidence of surgical site infection after emergency abdominal surgery in China
Source: Sci Rep. 2021 Apr 8;11:7794. doi: 10.1038/s41598-021-87392-8 (PMC8032698; doi:10.1038/s41598-021-87392-8)
Supplement: Supplementary file 2 — Supplementary Table 1. [file 41598_2021_87392_MOESM2_ESM.doc]

| **Table S2 The indications for surgery** | | |
| --- | --- | --- |
| Variables | Indications | Amount |
| Gastrointestinal surgery | gastrointestinal perforation | 161 |
|  | malignant intestinal obstruction | 97 |
|  | acute appendicitis | 568 |
|  | intestinal necrosis | 8 |
|  | toxic megacolon | 1 |
|  | gastrointestinal foreign body | 3 |
| Hepatobiliary surgery | acute cholecystitis | 53 |
|  | acute cholangitis | 3 |
|  | hepatic rupture | 9 |
| Others | abdominal organ injury due to trauma, | 6 |
|  | constrictive hernia | 34 |
|  | acute pancreatitis with infection | 4 |
