# Supplementary material for: Prospective multicenter study on the incidence of surgical site infection after emergency abdominal surgery in China
Source: Sci Rep. 2021 Apr 8;11:7794. doi: 10.1038/s41598-021-87392-8 (PMC8032698; doi:10.1038/s41598-021-87392-8)
Supplement: Supplementary file 3 — Supplementary Table 2. [file 41598_2021_87392_MOESM3_ESM.docx]

| Hospital | Number of included patients | Number of SSI patients(%) |
| --- | --- | --- |
| people’s hospital of Xinjiang uygur autonomous region | 89 | 16(18.0) |
| Shangqiu first people’s hospital | 72 | 8(11.1) |
| the affiliated hospital of Qingdao university | 64 | 4(6.3) |
| Yueyang first people's hospital | 43 | 2(4.7) |
| first affiliated hospital of Xinjiang medical university | 42 | 11(26.2) |
| Shoukang hospital | 42 | 3(7.1) |
| Dongguan kanghua hospital | 41 | 2(4.9) |
| Shengli oilfield central hospital of Shandong province | 40 | 2(5.0) |
| the 901th hospital of the PLA | 39 | 2(5.1) |
| the first people's hospital of Taizhou city | 35 | 1(2.9) |
| the people's hospital of Guangxi zhuang autonomous region | 32 | 2(6.3) |
| Zigong first people’s hospital | 30 | 2(6.7) |
| general hospital of dongfang hospital group | 29 | 1(3.4) |
| Tsinghua university affiliated Beijing Tsinghua chang gung hospital | 29 | 4(13.8) |
| the second hospital of Jilin university | 23 | 1(4.3) |
| Lanzhou general hospital of Lanzhou military | 22 |  |
| Chenzhou first people’s hospital | 22 | 1(4.5) |
| Shaoxing central hospital | 18 |  |
| northern Jiangsu people’s hospital | 23 | 1(4.3) |
| people's hospital of Dongying city | 16 |  |
| central war zone hospital of PLA | 15 |  |
| FAW general hospital of Jilin province | 15 |  |
| Zigong fourth people’s hospital | 14 | 1(7.1) |
| the second hospital, university of south China | 14 |  |
| Baotou central hospital | 13 | 1(7.7) |
| Langxi county traditional chinese medicine hospital | 12 | 1(8.3) |
| Nanyang city center hospital | 12 |  |
| Jinling hospital, affiliated to southeast university | 14 | 2(14.3) |
| Zhangjiagang first people’s hospital | 10 | 1(10.0) |
| Guangdong provincial people's hospital | 9 |  |
| Xishan people's hospital of Wuxi city | 8 |  |
| Heji hospital affiliated to Changzhi medical college | 7 |  |
| the affiliated hospital of Xuzhou medical university | 7 |  |
| Nanchong central hospital | 7 |  |
| the first affiliated hospital of Zhengzhou university | 6 | 1 |
| the second affiliated hospital of Dalian medical university | 6 |  |
| the 900th hospital of the PLA | 5 |  |
| Tianjin first central hospital | 5 |  |
| the second Xiangya hospital of central south university | 3 |  |
| Sir Run Run Shaw Hospital of Zhejiang University School of Medicine | 3 |  |
| general hospital of Tianjinmedical university | 3 |  |
| Liyang people's hospital | 3 |  |
| the first affiliated hospital of Kunming medical university | 3 |  |
| Hunan people’s hospital | 3 |  |
| Chinese PLA general hospital | 2 |  |
| West China Hospital | 2 | 1 |
| the affiliated hospital of Xuzhou medical university | 1 |  |
